# Birth weight is not causally associated with adult asthma: results from instrumental variable analyses

**DOI:** 10.1038/s41598-019-44114-5

**Published:** 2019-05-21

**Authors:** Ping Zeng, Xinghao Yu, Xiang Zhou

**Affiliations:** 1 Department of Epidemiology and Biostatistics, Xuzhou Medical University, Xuzhou, Jiangsu 221004 China; 2 Department of Biostatistics, University of Michigan, Ann Arbor, Michigan 48109 USA; 3 Center for Statistical Genetics, University of Michigan, Ann Arbor, Michigan 48109 USA

**Keywords:** Genetic association study, Medical genetics

## Abstract

The association between lower birth weight and childhood asthma is well established. However, it remains unclear whether the influence of lower birth weight on asthma can persist into adulthood. We conducted a Mendelian randomization analysis to assess the causal relationship of birth weight (~140,000 individuals) on the risk of adult asthma (~62,000 individuals). We estimated the causal effect of birth weight to be 1.00 (95% CI 0.98~1.03, *p* = 0.737) using the genetic risk score method. We did not observe nonlinear relationship or gender difference for the estimated causal effect. With the inverse-variance weighted method, the causal effect of birth weight on adult asthma was estimated to be 1.02 (95% CI 0.84~1.24, *p* = 0.813). Additionally, the iMAP method provides no additional genome-wide evidence supporting the causal effects of birth weight on adult asthma. Our results were robust against various sensitivity analyses, and MR-PRESSO and MR-Egger regression showed that no instrument outliers and no horizontal pleiotropy were likely to bias the results. Overall, our study provides no evidence for the fetal origins of diseases hypothesis for adult asthma, implying that the impact of birth weight on asthma in years of children and adolescents does not persist into adult and previous findings may be biased by confounders.

## Introduction

Asthma is a commonly complex chronic lung disease that is characterized by bronchoconstriction, airway hyper-responsiveness, mucus secretion and chronic inflammation^1^. Asthma represents a growing severe public health burden, affecting more than 300 million people and causing approximately 250,000 deaths per year worldwide^2,3^. Although asthma is very common in childhood, it can also occur in adulthood; for example, the incidence of asthma among adults is estimated to be as high as 12 cases per 1,000 person-years^4,5^. Childhood and adult asthma share the same disease symptoms but likely have different genetic and environmental causes^6–11^. The mechanism of asthma remains elusive. In the literature it has been reported that various environmental, familial, socioeconomic, lifestyle and genetic factors are associated with asthma (e.g. obesity, smoking, air pollution and allergies)^9,12–15^. Among these identified risk factors, the relationship between birth weight and subsequent risk of asthma has attracted much attention^16,17^. In practice birth weight is widely employed as a proxy measurement of early life development and has long been hypothesized to have a profound long-term impact on individual’s predisposition to the risk of various diseases (e.g. asthma^18^) in later life — a hypothesis often referred to as the Barker hypothesis of adult diseases, or the fetal origins of adult diseases^18–23^. Indeed, it has been previously found in observational studies that lower birth weight is correlated to higher risk of asthma in both childhood and adolescence^23–27^. More, importantly, this inverse association between birth weight and childhood asthma is unlikely confounded by familial factors^28^ and is also supported by large-scale meta-analyses^3,17,29,30^.

However, whether lower birth weight still has a long-term influence on the risk of adult asthma is less understood and only very few studies have previously focused on this question^17,31,32^. It was observed that the prevalence of asthma at 26 years among the lowest birth weight group (<2 kg) was about twice higher compared with the reference birth weight group (3~3.5 kg)^32^. Another finding reported in Johnson *et al*.^31^ implied that the asthmagenic effect of low birth weight can persist into adulthood. Additionally, a recent meta-analysis also showed that the risk of developing adult asthma for individuals born small (birth weight <2.5 kg) was 25% higher compared with those with normal birth weight (2.5~4.0 kg)^17^. Nevertheless, it remains unclear whether the inverse association between birth weight and adult asthma in those studies is truly causal as many known/unknown factors (e.g. smoking or body mass index) in later childhood or early adulthood can confound the observed relationship between birth weight and adult asthma^32^.

Understanding the long-term causal impact of birth weight on individual’s predisposition to asthma risk can facilitate our understanding of asthma etiology and paves ways for the potential development of early interventions to reduce asthma risk in adulthood. However, determining the causal impact of birth weight on adult asthma through traditional randomized intervention studies is a challenging task as such studies necessarily require a relatively long follow up, thus time-consuming and expensive, and are generally unethical to perform in practice^33,34^. Therefore, it is desirable to determine the causal relationship between birth weight and adult asthma in observational studies using other novel statistical strategies^35^. In the literature of causal inference, Mendelian randomization (MR) is a novel statistical approach that is commonly employed to determine the causal relationship between an exposure variable (e.g. birth weight) and an outcome variable (e.g. adult asthma) in observational studies. Specifically, MR is an instrumental variable method for causal inference that relies on strongly associated single nucleotide polymorphisms (SNPs) from genome-wide association studies (GWASs) to serve as instruments^36,37^. By leveraging the fact that the two alleles of a genetic variant are randomly segregated during gamete formation and conception under the Mendel’s law and that such segregation is independent of various environmental confounders, MR analysis can provide an estimate of causal effect without much susceptibility to reverse causation and other confounding factors as compared with other statistical approaches^38^.

In the present study we performed a MR study based on two causal inference approaches including genetic risk score and two-sample inverse-variance weighted (IVW) estimation. Our study employed summary statistics obtained from large-scale GWASs with sample sizes ranging up to ~140,000 individuals for birth weight and ~62,000 individuals for adult asthma, representing the largest MR analysis performed to date for inferring the causal relationship between birth weight and adult asthma. Even with such large sample sizes, however, our study did not provide sufficient statistical evidence that supports the causal role of birth weight on adult asthma, suggesting that the previously observed association between birth weight and adult asthma may be unlikely a direct causal relationship.

## Materials and Methods

### Data sources and selection of instrumental variables

We first obtained summary statistics of birth weight from the Early Growth Genetics (EGG) GWAS consortium study^39^. The EGG study is the largest GWAS formally published and performed to date on birth weight, which analyzed a total of 16,245,523 genotyped and imputed SNPs on up to 143,677 individuals of European ancestry. In this EGG study, an additive linear regression model was applied to analyze one genetic variant at a time to detect the SNP association with birth weight while properly controlling for gestational age and study-specific covariates whenever they were available^39^. With the EGG GWAS summary statistics, we yielded a set of 59 independent index SNPs that were strongly associated with offspring birth weight at the genome-wide significance level (*p* < 5.00E-8) to serve as instrumental variables (see extended data table one shown in Horikoshi *et al*.^39^ for full information).

In our MR analysis a potential confounder is the maternal effect — the portion of mother’s genetic effect on offspring birth weight mediated through various maternal behaviors during pregnancy or intrauterine environment^40^. To control for confounding due to the maternal effect, we excluded instrumental variables that exhibited potential maternal effects on birth weight using summary statistics from a recently published GWAS of maternal SNP effects on offspring birth weight^40^. This maternal GWAS study included 86,577 women and analyzed a total of 8,741,106 genotyped and imputed SNPs. While the sample size in the maternal GWAS is large, it is about half smaller compared with the offspring EGG GWAS (86,577 vs. 143,677). Therefore, to effectively remove all SNPs that may display observable maternal effects, we obtained a set of birth weight associated maternal SNPs in terms of a relaxed significance threshold (1.00E-5). Totally, we generated 700 SNPs which likely showed potential maternal effects. Afterwards, we then cross-examined the 59 instrumental variables with these maternal SNPs and removed instrumental variables that resided within 1 Mb of any of the maternal SNPs. By doing this, twelve instrumental variables were further excluded.

To minimize the influence of the potential pleiotropic effects, we also removed instrumental variables that were associated with relevant allergic diseases including asthma, hay fever and eczema. Specifically, we obtained summary results for these three allergic diseases from a recently published GWAS^13,14^ and yielded the corresponding p values of the selected instrumental variables for each disease. We then removed instrumental variables that may show potential associations with asthma, hay fever or eczema (*p* < 0.05/58 = 8.62E-4). Excluding instrumental variables that are strongly correlated to the outcome of interest (or outcome relevant traits) is a conservative strategy to guarantee the validity of the MR analysis — by focusing on only instrumental variables that do not have horizontal pleiotropic effects, we can ensure that these instrumental variables only have an influence on adult asthma by the path of birth weight^41–43^. Afterwards, two additional instrumental variables were excluded in this filtering step. We focused our following analysis on the remaining 45 instrumental variables that unlikely exhibit maternal effects and unlikely exhibit pleiotropic effects. We would further examine the possible influence of instrument pleiotropy in our sensitivity analyses (see below).

Next, we obtained asthma data from the Genetic Epidemiology Research on Aging (GERA) cohort^44^. The GERA study included adult individuals whose age ranged from 18 to over 100 years old (with an average age of 63 years at the time of the survey in 2007), indicating that all the individuals included in our analysis were adult. In the GERA study asthma was defined by the international classification of diseases (ninth revision with clinical modification; ICD-9-CM) in terms of the Kaiser Permanente Northern California patient electronic medical record (EMR). More specifically, an individual was coded with asthma if she/he had at least two diagnoses in the asthma category of ICD-9-CM recorded on separate days (i.e. 493, 493, 493, 493.01, 493.02, 493.1, 493.1, 493.11, 493.12, 493.2, 493.2, 493.21, 493.22, 493.8, 493.81, 493.82, 493.9, 493.9, 493.91 and 493.92). By this way, there were 10,101 (16.3%) adult asthma cases. Besides asthma disease information from EMR, we also obtained from survey multiple demographic and behavioral factors which included family income, education level, gender, alcohol and smoking statuses, body mass index (BMI) and general health status (descriptions of these factors are shown in Table 1).

**Table 1:** Descriptions of covariates available from the GERA cohort study.

Covariates	Code and proportion (%)
Education	0: Other (4.97); 1: Elementary, High School or Technical School (12.6); 2: Some college (23.3); 3: College/Graduate School (59.1)
Income	1: <$39,999 (16.0); 2: $40,000–$59,999 (16.6); 3: $60,000–$99,999 (30.3); 4: >$100,000 (37.1)
BMI	1: < = 18 (1.66); 2: 19–25 (44.6); 3: 26–29 (28.7); 4: 30–39 (22.0); 5: > 40 (3.00)
Gender	1: male (39.9); 2: female (60.1)
General health	1: Excellent (19.3); 2: Very Good (38.2); 3: Good (33.7); 4: Fair/Poor (8.74)
Smoking	0: 0 pack years (57.2); 1: < = 10 pack years (14.9); 2: 10~20 pack years (15.3); 3: 20~30 pack years (8.76); 4: > 30 pack years (3.85)
Alcohol	1: no days (35.6); 2: 1 day (14.3); 3: 2–4 days (20.8); 4: 5–6 days (10.5); 5: every day (18.8)

Note: GERA: Genetic Epidemiology Research on Aging; BMI: body mass index; all the covariates were incorporated into the logistic regression as continuous variables.

After proper quality control [Hardy-Weinberg equilibrium (HWE) test *p* value < 10^−4^, genotype call rate < 95% and minor allele frequency (MAF) < 0.01], we left a total of 487,609 SNPs on 61,916 (24,718 males and 37,198 females) individuals of European ancestry. To yield genotypes of instruments from the GERA cohort, we phased genotypes using SHAPEIT^45^ and imputed SNPs based on the Haplotype Reference Consortium (HRC version r1.1) reference panel^46^ on the Michigan Imputation Server using Minimac3^47^. After filtering (HWE *p* value < 10^−4^, genotype call rate < 95%, MAF < 0.01 and imputation score < 0.30), we obtained 8,385,867 genotyped and imputed SNPs. For each genotyped or imputed SNP in the GERA cohort, we generated association results for adult asthma by using an additive logistic regression model while controlling for other available covariates (e.g. top ten principal components and those factors presented in Table 1). Note that, among the set of 45 instrumental variables for birth weight, only 37 were available after filtering and imputation. For each of the remaining instrumental variables in turn, we obtained summary statistics for both birth weight and adult asthma in terms of effect allele, marginal effect size, and standard error as well as p value (Table 2).

**Table 2:** Summary statistics information for the selected instrument variables of birth weight and adult asthma.

Chr	SNP	Position	A_1_/A_2_	Birth weight			Adult asthma		
				beta	se	*p*	beta	se	*p*
1	rs2473248	22,536,643	C/T	0.0325	0.0057	1.00E-08	0.0050	0.0224	0.823
1	rs3753639	154,986,091	C/T	0.0306	0.0045	7.30E-12	−0.0175	0.0180	0.331
1	rs72480273	161,644,871	C/A	0.0313	0.0051	8.00E-10	0.0090	0.0205	0.661
1	rs61830764	212,289,976	A/G	0.0220	0.0040	5.60E-08	0.0020	0.0147	0.892
2	rs1374204	46,484,205	T/C	0.0470	0.0042	6.20E-29	−0.0326	0.0169	0.054
3	rs11719201	123,068,744	T/C	0.0463	0.0044	2.40E-26	0.0109	0.0181	0.547
3	rs10935733	148,622,968	T/C	0.0221	0.0039	9.20E-09	−0.0067	0.0158	0.672
4	rs925098	17,919,811	G/A	0.0340	0.0042	5.40E-16	−0.0079	0.0175	0.652
5	rs854037	57,091,783	A/G	0.0268	0.0048	2.20E-08	−0.0045	0.0201	0.823
5	rs7729301	157,886,953	A/G	0.0239	0.0042	1.60E-08	−0.0068	0.0175	0.698
6	rs35261542	20,675,792	C/A	0.0444	0.0041	4.40E-27	0.0218	0.0173	0.208
6	rs7742369	34,165,721	G/A	0.0283	0.0049	9.90E-09	−0.0075	0.0201	0.709
7	rs798489	2,801,803	C/T	0.0233	0.0042	2.00E-08	−0.0254	0.0173	0.142
7	rs6959887	35,295,365	A/G	0.0228	0.0038	1.50E-09	0.0070	0.0150	0.641
8	rs13266210	41,533,514	A/G	0.0308	0.0045	1.30E-11	0.0237	0.0183	0.195
8	rs6989280	126,508,746	G/A	0.0218	0.0042	2.20E-07	0.0139	0.0175	0.427
8	rs12543725	142,247,979	G/A	0.0231	0.0038	1.20E-09	−0.0172	0.0157	0.273
9	rs28510415	98,245,026	G/A	0.0557	0.0065	1.50E-17	−0.0337	0.0269	0.210
9	rs2150052	113,945,067	T/A	0.0211	0.0038	2.20E-08	0.0109	0.0151	0.470
9	rs700059	125,824,055	G/A	0.0334	0.0054	4.70E-10	0.0080	0.0243	0.742
10	rs61862780	94,468,643	T/C	0.0281	0.0037	3.00E-14	−0.0274	0.0153	0.073
10	rs74233809	104,913,940	C/T	0.0366	0.0067	5.20E-08	−0.0016	0.0262	0.951
10	rs2421016	124,167,512	T/C	0.0207	0.0037	1.80E-08	0.0080	0.0154	0.603
11	rs72851023	2,130,620	T/C	0.0476	0.0075	2.90E-10	−0.0491	0.0338	0.146
12	rs12823128	26,872,730	T/C	0.0211	0.0037	1.90E-08	0.0188	0.0155	0.225
12	rs1351394	66,351,826	T/C	0.0436	0.0037	1.90E-32	−0.0151	0.0154	0.327
12	rs7964361	102,994,878	A/G	0.0391	0.0067	4.70E-09	−0.0186	0.0274	0.497
13	rs2324499	40,662,001	G/C	0.0217	0.0040	7.30E-08	0.0178	0.0160	0.266
13	rs2854355	48,882,363	G/A	0.0234	0.0044	9.80E-08	0.0119	0.0171	0.486
13	rs1819436	78,580,283	C/T	0.0329	0.0057	6.30E-09	−0.0082	0.0229	0.720
16	rs1011939	19,992,996	G/A	0.0217	0.0041	1.30E-07	−0.0411	0.0172	0.017
17	rs113086489	7,171,356	T/C	0.0307	0.0038	9.10E-16	−0.0479	0.0155	0.002
19	rs10402712	33,926,013	A/G	0.0215	0.0043	4.40E-07	0.0218	0.0176	0.215
20	rs6040076	10,658,882	C/G	0.0231	0.0039	2.00E-09	−0.0215	0.0154	0.163
20	rs6016377	39,172,728	T/C	0.0239	0.0039	9.50E-10	0.0060	0.0148	0.685
21	rs2229742	16,339,172	G/C	0.0360	0.0060	2.20E-09	0.0276	0.0254	0.277
22	rs134594	29,468,456	C/T	0.0227	0.0040	1.00E-08	0.0257	0.0161	0.110

Note: Chr represents the chromosome; A_1_ is the effect allele and A_2_ is the alterative allele.

### Genetic risk score method

The genetic risk score (GRS) for birth weight was computed following previous studies^48,49^. Briefly, the GRS for individual *i* in the GERA study was constructed as

1 $${{\rm{GRS}}}_{i}=\sum _{j=1}^{37}SN{P}_{ij}{\hat{\beta }}_{j}^{{\rm{birthweight}}},$$

where ${\hat{\beta }}_{j}^{{\rm{birthweight}}}$ is the estimated marginal SNP effect size of birth weight for the *j*th instrumental variable obtained from the EGG study^39^, and *SNP*_*ij*_ is the individual-level genotype of the corresponding *j*th instrumental in the GERA study^44^ and was coded to be 0, 1 and 2 in terms of the number of the effect allele which was matched with that in the EGG study. We further standardized GRS to have mean zero and variance one in our analysis. Note that, unlike in^48,49^ we did not scale GRS as the p value of GRS would not change regardless GRS was scaled or not. Afterwards, we evaluated the effect of GRS on adult asthma with an additive logistic regression model while adjusting for available covariates (see Table 1) as well as top ten genotype principal components

2 $$\mathrm{log}\,{\rm{it}}({\mu }_{i})={{\rm{GRS}}}_{i}\theta +{{\boldsymbol{X}}}_{i}^{T}{\boldsymbol{\alpha }},$$

where *μ*_*i*_ is the expectation of *y*_*i*_ with *y*_*i*_ = 1 or 0 representing the status of adult individual *i* with or without asthma in the GERA study, *θ* is the effect size of GRS, and ***X***_*i*_ is the vector of covariates with effect sizes ***α***. We are primarily interested in estimating *θ* and testing for the null hypothesis *H*_0_: *θ* = 0.

### Two-sample MR analysis

Besides the genetic risk score method, we also performed a two-sample MR analysis to estimate the causal effect size of birth weight on adult asthma using summary statistics (Table 2). Suppose that the effect size estimate and its variance for the *j*th instrumental variable of birth weight are ${\hat{\beta }}_{j}^{{\rm{birthweight}}}$ and ${\rm{var}}({\hat{\beta }}_{j}^{{\rm{birthweight}}})$ (*j* = 1, 2, …, 37), both of which were obtained from the EGG study^39^. Suppose ${\hat{\beta }}_{j}^{{\rm{adult}}\text{asthma}}$ and ${\rm{var}}({\hat{\beta }}_{j}^{{\rm{adult}}\text{asthma}})$ are the effect size estimate and its variance for the same instrumental variable for adult asthma in the GERA study^44^, respectively. We estimated the causal effect of birth weight (again, denoted as *θ*) using all the instrumental variables together through the IVW method^50–55^

3 $$\begin{array}{rcl}\hat{\theta } & = & \frac{{\sum }_{j=1}^{37}{\rm{var}}{({\hat{\beta }}_{j}^{{\rm{adult}}\text{asthma}})}^{-1}{\hat{\beta }}_{j}^{{\rm{adult}}\text{asthma}}{\hat{\beta }}_{j}^{{\rm{birthweight}}}}{{\sum }_{j=1}^{37}{\rm{var}}{({\hat{\beta }}_{j}^{{\rm{adult}}\text{asthma}})}^{-1}{({\hat{\beta }}_{j}^{{\rm{birthweight}}})}^{2}},\\ {\rm{var}}(\hat{\theta }) & = & \frac{1}{{\sum }_{j=1}^{37}{\rm{var}}{({\hat{\beta }}_{j}^{{\rm{adult}}\text{asthma}})}^{-1}{({\hat{\beta }}_{j}^{{\rm{birthweight}}})}^{2}}.\end{array}$$

### The iMAP analysis to infer the causal effect

We further applied a recently developed method, iMAP, to complementally analyze the relationship between birth weight and adult asthma. iMAP is an integrative method for modeling pleiotropy and can be employed to investigate causality between pairs of complex traits using summary statistics from GWAS^56^. Unlike the genetic score or the two-sample MR method, iMAP jointly analyzes all genome-wide SNPs and has the potential to provide additional evidence supporting or against causal relationship between two traits. iMAP aims to estimate some proportion parameters that characterize the SNP causal effects on the two traits in order to better understand the relationship between the two traits^56^. In particular, iMAP estimates an important ratio quantity π_11_/(π_10_ + π_11_) (or π_11_/(π_01_ + π_11_)), where *π*_11_ represents the probability that a SNP is associated with both traits, *π*_10_ represents the probability that a SNP is associated with the first trait but not the second, *π*_01_ represents the probability that a SNP is associated with the second trait but not the first and *π*_00_ represents the probability that a SNP is not associated with any traits. Therefore, this calculated quantity above represents the proportion of SNPs associated with one trait that are also associated with the other and has been employed to evaluate the causality of one trait on the other^57^. Specifically, a large π_11_/(π_10_ + π_11_) and a small π_11_/(π_01_ + π_11_) imply that a large fraction of SNPs associated with the first trait is also associated with the second trait, but not vice versa, indicating that the first trait may causally affect the second trait. A small π_11_/(π_10_ + π_11_) and a large π_11_/(π_01_ + π_11_) indicate that the second trait may causally affect the first trait. On the other hand, a large π_11_/(π_10_ + π_11_) and a large π_11_/(π_01_ + π_11_) indicate that both traits may share common biological pathways. Therefore, estimating π_11_/(π_10_ + π_11_) and π_11_/(π_01_ + π_11_) using iMAP can help provide additional evidence with regard to the causal relationship between birth weight and adult asthma.

### Sensitivity analyses

To ensure the robustness of our results and to guard against various modeling misspecifications in our main Mendelian mediation analyses, we performed extensive selectivity analyses. First, to further examine the pleiotropic effects of instruments, we searched the NHGRI-EBI catalog to look at whether there were instrumental variables that may have any associations with other traits or diseases. We found that twelve instrumental variables were previously identified to be associated with other traits or diseases (Tables 3 and 4). We then carried out a leave-one-out (LOO) analysis to check if removing any of these twelve SNPs could substantially influence the results of genetic risk score and MR. In addition, for the genetic risk score approach, we carried out stratified analysis in terms of gender. For the two-sample MR method, we conducted the Mendelian randomization pleiotropy residual sum and outlier (MR-PRESSO) method to identify instrumental outliers that can substantially influence the causal effect estimate^58^. We also conducted weighted median-based method which is robust when some instrumental variable are invalid^59^ as well as MR-Egger regression which guards against horizontal pleiotropic effects^60,61^.

**Table 3:** Leave-one-out analysis of genetic risk score by removing instrumental variables of birth weight that were associated with other traits or diseases.

SNP	Chr	Mapped gene	Traits/diseases	*p*	PubMed ID	LOO analysis
						OR (95% CI and *p* value)
rs11719201	3	*ADCY5*	heel bone mineral density	5.00E-11	30048462	1.00 (0.97~1.02, *p* = 0.847)
rs35261542	6	*CDKAL1*	type 2 diabetes	2.00E-14	29358691	0.99 (0.97~1.01, *p* = 0.467)
rs35261542	6	*CDKAL1*	hemoglobin A1c levels	2.00E-34	29403010	0.99 (0.97~1.01, *p* = 0.467)
rs35261542	6	*CDKAL1*	BMI	4.00E-29	28892062	0.99 (0.97~1.01, *p* = 0.467)
rs7742369	6	*HMGA1*	height	1.00E-13	20189936	0.99 (0.97~1.02, *p* = 0.648)
rs798489	7	*AMZ1*	waist circumference adjusted for BMI	2.00E-08	28448500	1.00 (0.97~1.02, *p* = 0.791)
rs2150052	9	*RP11–202G18.1*	neutrophil percentage of white cells	3.00E-10	27863252	1.00 (0.97~1.02, *p* = 0.702)
rs2421016	10	*PLEKHA1*	type 2 diabetes	4.00E-11	28869590	1.00 (0.97~1.02, *p* = 0.791)
rs1351394	12	*HMGA2*	hip circumference adjusted for BMI	5.00E-13	25673412	1.00 (0.97~1.02, *p* = 0.734)
rs1351394	12	*HMGA2*	height	2.00E-65	20881960	1.00 (0.97~1.02, *p* = 0.734)
rs1351394	12	*HMGA2*	birth length	7.00E-07	25281659	1.00 (0.97~1.02, *p* = 0.734)
rs6040076	20	*intergenic*	pulse pressure	9.00E-18	28739976	1.00 (0.98~1.02, *p* = 0.940)
rs2229742	21	*NRIP1*	spherical equivalent or myopia (age of diagnosis)	9.00E-10	27197191	0.99 (0.97~1.02, *p* = 0.604)
rs6989280	8	*TRIB1*	childhood BMI	3.10E-10	26604143	1.00 (0.97~1.02, *p* = 0.744)
rs1011939	16	*GPR139*	childhood BMI	4.30E-10	26604143	1.00 (0.97~1.01, *p* = 0.479)
rs10402712	19	*PEPD*	childhood obesity	3.6λ-06	22484627	1.00 (0.97~1.02, *p* = 0.789)

Note: BMI: body mass index. We also searched the GWAS catalog (https://www.ebi.ac.uk/gwas; until 21/12/2018) to check if there were instrumental variables which had any associations with other traits or diseases. During the search we paid special attention to two birth-weight related early growth traits (i.e. childhood BMI and childhood obesity). We identified SNPs which resided within 1 Mb of any of the instruments variables of birth weight and may be potentially associated with these two traits in terms of their summary statistics results (*p* < 1E-5). Finally, we found that twelve instrumental variables were previously identified to be associated with other traits or diseases. We then carried out a leave-one-out (LOO) analysis to check if removing any of these twelve SNPs could substantially influence the results of genetic risk score (last column). If removing all the twelve instrumental variables, the causal effect was estimated to be 0.99 (95% CI 0.97~1.01, *p* = 0.417).

**Table 4:** Leave-one-out analysis of MR by removing instrumental variables of birth weight that were associated with other traits or diseases.

SNP	Chr	Mapped gene	Traits/diseases	*p*	PubMed ID	LOO analysis
						OR (95% CI and *p* value)
rs11719201	3	*ADCY5*	heel bone mineral density	5.00E-11	30048462	0.99 (0.81~1.21, *p* = 0.931)
rs35261542	6	*CDKAL1*	type 2 diabetes	2.00E-14	29358691	0.94 (0.77~1.15, *p* = 0.567)
rs35261542	6	*CDKAL1*	hemoglobin A1c levels	2.00E-34	29403010	0.94 (0.77~1.15, *p* = 0.567)
rs35261542	6	*CDKAL1*	BMI	4.00E-29	28892062	0.94 (0.77~1.15, *p* = 0.567)
rs7742369	6	*HMGA1*	height	1.00E-13	20189936	0.97 (0.80~1.18, *p* = 0.771)
rs798489	7	*AMZ1*	waist circumference adjusted for BMI	2.00E-08	28448500	1.00 (0.82~1.21, *p* = 0.966)
rs2150052	9	*RP11–202G18.1*	neutrophil percentage of white cells	3.00E-10	27863252	0.97 (0.80~1.17, *p* = 0.733)
rs2421016	10	*PLEKHA1*	type 2 diabetes	4.00E-11	28869590	0.98 (0.81~1.19, *p* = 0.865)
rs1351394	12	*HMGA2*	hip circumference adjusted for BMI	5.00E-13	25673412	0.95 (0.77~1.16, *p* = 0.594)
rs1351394	12	*HMGA2*	height	2.00E-65	20881960	0.95 (0.77~1.16, *p* = 0.594)
rs1351394	12	*HMGA2*	birth length	7.00E-07	25281659	0.95 (0.77~1.16, *p* = 0.594)
rs6040076	20	*intergenic*	pulse pressure	9.00E-18	28739976	1.00 (0.82~1.21, *p* = 0.975)
rs2229742	21	*NRIP1*	spherical equivalent or myopia (age of diagnosis)	9.00E-10	27197191	0.96 (0.79~1.17, *p* = 0.694)
rs6989280	8	*TRIB1*	childhood BMI	3.10E-10	26604143	0.97 (0.80~1.17, *p* = 0.736)
rs1011939	16	*GPR139*	childhood BMI	4.30E-10	26604143	0.95 (0.78~1.15, *p* = 0.590)
rs10402712	19	*PEPD*	childhood obesity	3.60E-06	22484627	0.95 (0.82~1.20, *p* = 0.929)

Note: BMI: body mass index. We also searched the GWAS catalog (https://www.ebi.ac.uk/gwas; until 21/12/2018) to check if there were instrumental variables which had any associations with other traits or diseases. During the search we paid special attention to two birth-weight related early growth traits (i.e. childhood BMI and childhood obesity). We identified SNPs which resided within 1 Mb of any of the instruments variables of birth weight and may be potentially associated with these two traits in terms of their summary statistics results (*p* < 1E-5). Finally, we found that twelve instrumental variables were previously identified to be associated with other traits or diseases. We then carried out a leave-one-out (LOO) analysis to check if removing any of these twelve SNPs could substantially influence the results of MR (last column). If removing all the twelve instrumental variables, the causal effect was estimated to be 0.89 (0.70~1.13, p = 0.343).

### Power calculation

Finally, to investigate the statistical power, we carried out power calculation to detect a non-zero causal effect for birth weight with regard to adult asthma^62–64^. In the calculation, we set the total phenotypic variance explained (PVE) by all instrumental variables to be 1.23% (i.e. the total phenotypic variance of birth weight explained by all used instrumental variables; see below), set the significance level *α* to be 0.05, and set the proportion of the asthma cases to be 16.3% (i.e. the fraction of cases observed in the GERA study). In the present study, the power was calculated using the method shown in Brion *et al*.^63^.

### Ethical approval and informed consent

Our study made use of data generated in previous studies, in which individuals gave informed consent for data sharing, as described in each of the GWASs used in the present manuscript. Additional ethical approval was also not needed for our study.

## Results

### Estimated causal effect of birth weight on asthma with genetic risk score

We employed a set of 37 SNPs from a large-scale GWAS with up to 143,677 European individuals to serve as valid instrumental variables for offspring birth weight (Table 1). These SNPs are all robustly associated with birth weight (*p* < 5.00E-8)^39^, and explain a total of 1.23% phenotypic variance of birth weight based on summary statistics. We first examined the strength of these instrumental variables using *F* statistic^65^. The *F* statistics for all these selected SNPs are above 10 (ranging from 25.0 to 138.9 with an average of 46.7), suggesting that all the instrumental variables are strong and that weak instrument bias unlikely occurs in our analysis.

Using the logistic regression, we find that no causal association exists between the genetically determined birth weight and adult asthma. Specifically, the odds ratio (OR) per risk score unit change is 1.00 [95% confidence interval (CI) 0.98~1.03, *p* = 0.737] after adjusting for covariates, with the unadjusted OR estimated to be 1.00 (95% CI 0.98~1.02, *p* = 0.816). In addition, there is no evidence for the quadratic effect of GRS (*p* = 0.602). We further implemented stratified logistic analysis in terms of gender. The OR is 0.97 (95% CI 0.93~1.01, *p* = 0.108) for men; while the OR is 1.02 (95% CI 0.99~1.05, *p* = 0.118) for women. No quadratic effect of GRS on adult asthma is detected in either men (*p* = 0.846) or women (*p* = 0.436). Additionally, the LOO analysis shows that none of the twelve instrumental variables that were previously identified to be associated with other traits or diseases can substantially change the estimated casual effect of GRS (Table 3).

### Estimated causal effect of birth weight on asthma with two-sample IVW method

**Figure 1:**
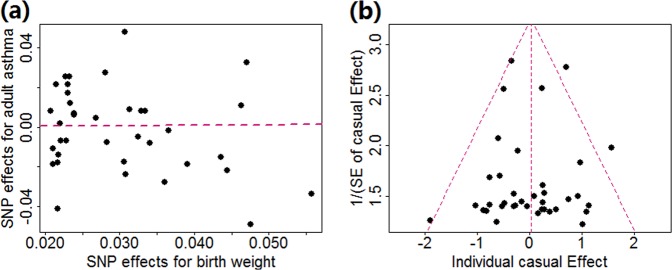
(**a**) Relationship between the SNP effect size estimates of birth weight (x-axis) and the corresponding effect size estimates of adult asthma (y-axis) using 37 instrument variables. The line in red represents the estimated casual effect of birth weight on adult asthma obtained using the IVW method. (**b**) Funnel plot for single causal effect estimate of birth weight on adult asthma. The vertical line in red represents the estimated casual effect of birth weight on adult asthma obtained using the IVW method.

In terms of the two-sample IVW method, no evidence of heterogeneous casual effect of individual instrumental variable is observed (*p* = 0.078) and the OR per unit standard deviation change of offspring birth weight on adult asthma is 1.02 (95% CI 0.84~1.24, *p* = 0.813), again, implying that there is no causal association between birth weight and adult asthma. The SNP effect size of birth weight against the SNP effect size of adult asthma for each instrumental variable is shown in Fig. 1a. The weighted median method shows consistent null estimate (OR = 0.91, 95% CI 0.67~1.23, *p* = 0.533) and the MR-Egger regression also generates similar null estimate (OR = 0.78, 95% CI 0.36~1.72, *p* = 0.540). The intercept of the MR-Egger regression is not significantly deviated from zero and is estimated to be 0.008 (95% CI −0.015~0.032, *p* = 0.483), suggesting that the assumption of balanced pleiotropy holds in our two-sample MR analysis. MR-PRESSO shows that no outliers can substantially influence the casual effect estimate at the significance level of 0.05. The funnel plot for individual causal effect size estimated for each single instrumental variable demonstrates a symmetric pattern of effect size variation around the point estimate (Fig. 1b). Together, the MR-PRESSO test, the MR-Egger regression intercept and the funnel plot indicate that horizontal pleiotropy unlikely biases our results. Again, the LOO analysis demonstrates that none of the twelve instrumental variables that were previously identified to be associated with other traits or diseases can substantially influence the estimated casual effect of MR (Table 4).

### Results of the iMAP method

**Figure 2:**
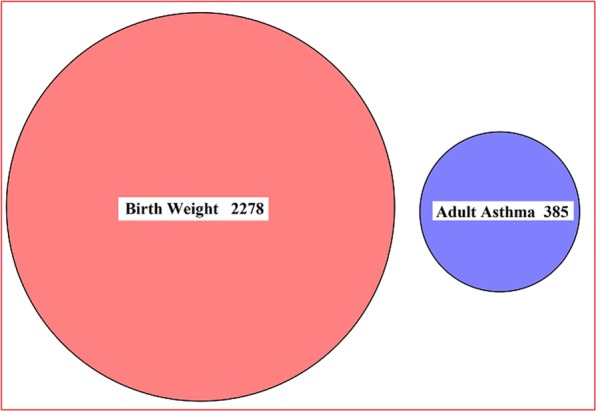
Venn diagram displays the association pattern of genome-wide significant SNPs (*p* < 5E-8) shared by birth weight and adult asthma. There are 2,278 and 385 SNPs which ware identified to be associated with birth weight and adult asthma, respectively; while no significant SNPs are shared by birth weight and adult asthma.

Using the iMAP method^56^, the proportion of SNPs associated with birth weight which are also associated with adult asthma is estimated to be 7.43E-4, the proportion of SNPs associated with adult asthma that are also associated with birth weight is 6.59E-5. Both the proportions are rather small and close to zero, suggesting that SNPs associated with the birth weight are unlikely to be associated with adult asthma. The result of iMAP is consistent with the observation that no association signals are overlapped between birth weight and adult asthma (Fig. 2). Additionally, the overall genetic correlation is only 0.050 (se = 0.069, *p* = 0.471) using the linkage disequilibrium score regression (LDSC)^39,66^. Therefore, both iMAP and LDSC provide no additional genome-wide evidence supporting the causal effects of birth weight on adult asthma.

### Results of power calculation

**Figure 3:**
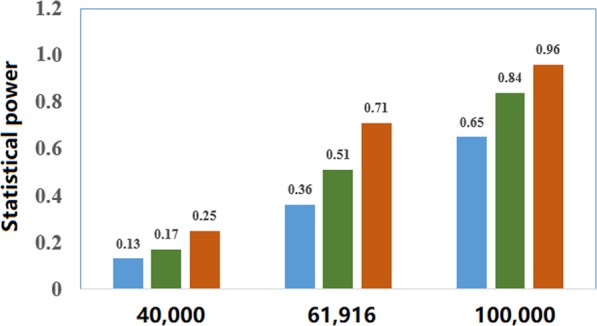
Statistical power calculation for the present MR analysis using the analytic method shown in^63^ (https://cnsgenomics.shinyapps.io/mRnd/). In the calculation, the total phenotypic variance explained by instrumental variables was set to be 1.23%, the significance level α was set to be 0.05, the proportion of the asthma cases was set to be 16.3%. Various sample sizes (i.e. 40,000, 61,916 and 100,000) were considered. For each situation of sample size, the OR was assumed to be 1.10, 1.20 or 1.30, respectively. The estimated power was shown on the top of each bar.

We finally examine whether the lack of detectable non-zero causal effect of birth weight on adult asthma is due to a lack of statistical power. To do so, supposing various sample sizes (i.e. 40,000, 61,916 and 100,000), we performed the statistical power calculation to detect an OR of 1.10, 1.20 or 1.30 in the risk of adult asthma per unit change of birth weight following the approach shown in^63^. Note that, these assumed ORs are approximately equal to the observed effect of birth weight on adult asthma in previous observational studies^17^. The results imply that we would have a moderate to high power to detect the causal association between birth weight and adult asthma (Fig. 3). For example, for the current sample size in the GERA study (i.e. assume the sample size of adult asthma is 61,916 and OR = 1.10, 1.20 or 1.30 in the power calculation), the estimated statistical power is 17%, 51% or 84%, respectively.

## Discussion

In the present paper we have explored the fetal origins of adult asthma hypothesis by performing a comprehensive Mendelian randomization analysis to investigate the causal effects of birth weight on adult asthma. To efficiently avoid possible violation of model assumptions, we have carefully chosen SNPs to serve as valid instrument variables and conducted extensive sensitivity analyses to ensure the validity of Mendelian randomization analysis^65,67^. With valid instrument variables from large scale GWAS of birth weight we have demonstrated that the genetically increased/decreased birth weight is not casually associated with adult asthma.

Our results are in contrast with previous associations between birth weight and asthma discovered in observational studies. However, the associations between birth weight and adult asthma in these previous observation studies may be confounded by many known/unknown confounders that occur during prenatal or postnatal life (e.g. the adult body mass index, BMI, and smoking status in adulthood)^27,32^. Therefore, the association previously detected in observational studies could be spurious associations. Indeed, by using a propensity score approach to control for confounders, it has been showed that birth weight is not associated with the risk of asthma during the first six years of life^68^. In addition, after considering the maternal smoking status in pregnancy^27^ and gestational age^26,69^, the estimated association size between lower birth weight with asthma is much reduced. Therefore, our MR results are consistent with these observational studies that properly controlled for confounding effects, providing additional evidence supporting that birth weight may not be directly associated with adult asthma.

Finally, we emphasize that we cannot completely rule out the possibility that we are underpowered to discover a weak causal influence of birth weight on adult asthma as shown in the power calculation (Fig. 3). A comprehensive investigation that can completely elaborate this issue requires dataset of adult asthma with larger sample size in the future. We also note that two large scale GWASs about adult asthma were published recently and the corresponding summary statistics results can be publicly available^13,14^. However, due to the following reasons, we did not consider either of these two datasets. In particular, in the study of Ferreira *et al*.^14^, the analysis was performed on three allergic diseases (i.e. asthma, hay fever and eczema elucidates), thus the asthma-specific summary statistics results cannot be obtained. Additionally, about half samples in the birth weight EGG study and about 40% individuals in Ferreira *et al*.^14^ came from the same UK BioBank data resource^70^, leading to the issue of sample overlap. Participant overlap in the MR analysis can result in severely biased causal effect estimates and the adjustment of sample overlap is statistically challenging^71^. For the study of Demenais *et al*.^13^, the summary statistics of adult asthma are available for only 16 instrument variables (vs 37 in the GERA study). The smaller number of instrument variables may result in a substantial loss of information and potentially lead to weak instrument bias. Indeed, using those 16 available instrument variables of birth weight from the EGG study^39^ and their corresponding summary statistics of adult asthma from Demenais *et al*.^13^, we obtained a similar null estimate of causal effect for birth weight on adult asthma (OR = 1.05, 95% CI 0.82~1.34, *p* = 0.701), again supporting our conclusions above.

## Conclusions

Overall, our results do not provide any evidence supporting for the fetal origins of diseases hypothesis for adult asthma, implying that the impact of birth weight on asthma is less possible to last into adult and that some of the previous findings on the association between birth weight and adult asthma may be biased by confounders.

## Data Availability

The summary data for the EGG GWAS consortium study can be available at http://egg-consortium.org/. The GERA cohort can be available by application to https://www.ncbi.nlm.nih.gov (with dbGaP study accession no phs000674.v1.p1). The NHGRI-EBI catalog of published GWASs can be available https://www.ebi.ac.uk/gwas.
